# Strategies for reducing regulatory barriers to focused ultrasound technology

**DOI:** 10.1186/2050-5736-3-S1-P59

**Published:** 2015-06-30

**Authors:** Liz Dobrenz

**Affiliations:** 1 Focused Ultrasound Foundation, Charlottesville, Virginia, United States

## Background/introduction

The U.S. Food and Drug Administration (FDA) has automatically classified focused ultrasound (FUS) devices Class III, which requires sponsors to demonstrate the safety and effectiveness of a device through clinical trials. This requirement adds significant time and cost to the device development process; reclassification to Class II would be highly beneficial for the future of FUS.

## Methods

Statute outlines two strategies for reclassifying FUS to a class with lower regulatory standards: filing a petition for reclassification based on new information and filing a de novo application to request a risk-based review of a novel technology.

## Results and conclusions

There are three additional options for Focused Ultrasound Foundation action with less direct effects on reclassification: creating grants for regulatory science, educating stakeholders about FDA, and increasing direct-to-FDA advocacy. In this paper, each of these options and alternatives is assessed based on its likelihood of achieving success, time to success, and cost. Ultimately, this analysis recommends that the Focused Ultrasound Foundation take no statutory action to reclassify FUS at this time, and initiate efforts to increase Foundation stakeholder understanding of FDA regulations and their impact on the future of FUS.

